# On Chloroform as an Emmenagogue

**Published:** 1852-07

**Authors:** David H. Gibson

**Affiliations:** Fort Towson, Choctaw Nation


*On Chloroform as an Emmenagogue.* By DAVID H. GIBSON, M. D., Fort Towson, Choctaw Nation.

Having nowhere seen, in the course of my professional reading, any allusions made to the use of chloroform, as an emmenagogue, I am induced to submit the following facts for publication, partly from a desire that relief may be afforded to the suffering, and partly from a sense of professional duty.

Cases I. II.—Occurring in the same person. In October last, Mrs. W——, having a violent headache, to obtain relief resorted to the inhalation of chloroform. Within an hour after the inhalation (which was but for a few seconds) she was flowing freely, and continued thus for *four days.* There was no irregularity of the function of menstruation in the succeeding month (Nov.), but another attack of headache supervening, she again had recourse to the chloroform, and in a half hour the menstrual secretion made its appearance, the discharge continuing for *five* days. In both instances, the chloroform was inhaled about ten days after the subsidence of her regular periods. Since the last inhalation, she has menstruated at her usual period. Mrs. W—— is slightly inclined to plethora, general health usually good, aged thirty-five years.

Case III.—In the absence of Mrs. W——, from home, her servant girl, having gotten hold of the chloroform, imitated Mrs. W.'s example. A like result was produced upon the girl, who menstruated for four days. The girl is very healthy and about thirty years of age. The inhalation was never renewed by her. In this case, the chloroform was inhaled two weeks prior to her usual period, at which time she again menstruated. Since then she has menstruated regularly.

Case IV.—Miss ——, aged 19—general health excellent—no deviation having ever taken place since her first menstrual period, was, during a visit to Mrs. ——, induced to inhale chloroform, through curiosity to experience the sensations produced by it. In a half hour the menstrual fluid made its appearance and the flow continued for four days. The inhalation in this instance was ten days antecedent to the regular period, with which it did not interfere. Mrs. W——, my informant in regard to the foregoing cases, is an intelligent and reliable lady.

Case V.—Came under my immediate observation. Was called to see Mrs. H——, found her suffering much from suppressed menstruation. To relieve urgent pain, ordered hot hip-bath, from which the patient experienced much relief. Waited three hours after the use of the bath, without recourse to any other means, having decided, as this was an opportune case, to exhibit the chloroform, which was done for *thirty* seconds. In *twenty* minutes after its administration, the patient was flowing freely and continued to do so for three days. Patient is of a weakly constitution, the result of much hardship. Age of the patient, about forty years. This case is the more remarkable from the fact that the patient has not menstruated for more than *eight* months.

The suppression was induced by causes not deemed necessary to relate at present. Prior to the suppression, she had been very regular for many years. Pregnancy has nothing to with the case, as the patient is not at this time, nor for many years past has she been in that condition.

I regret that I have not a greater number of cases to submit for the consideration of the profession. Being but a young practitioner, I am desirous that more experienced physicians should give the chloroform a trial, in order more fully, than my position will allow, to test its value as an emmenagogue; and diffident of my ability to account *correctly* for the "modus operandi" of the chloroform in the above cases, I shall without comment submit them to those who have better opportunities for investigation.

Before closing, however, I will present the following case. Miss——, aged 18 years, had an acute suppression of the menses, upon the first recurrence of the monthly period, subsequent to the age of puberty. Epileptic spasms quickly succeeded the suppression. Three years have elapsed since, and she is yet subject to these spasms, at longer or shorter intervals.

Within the last eight months, she was placed under my care. At one time, she will menstruate healthily—at another, there will not be the slightest appearance of menstruation—again, a leucorrhœa will take the place of the proper menstrual fluid. Sometimes, the leucorrhœal, as also the menstrual discharge, whichever it may be, will appear a week, sometimes two weeks antecedent to the regular period. Without entering into detail, as regards the treatment adopted in this case, I will submit the subjoined query. Upon the hypothesis, that the chloroform did act as an emmenagogue in the cases already related, how far would it comport with the safety of this epileptic patient to administer the chloroform? This question is predicated upon the belief, that, if the menstrual function were regularly performed, recovery from the epilepsy *might* take place. It is intended that the question shall refer more particularly to the epileptic condition, than to the irregularity of the menstrual function. I have no reason to suppose the existence of organic disease, either of the brain or uterus, in this patient.

